# Effects of Lysophospholipid on Growth Performance, Hepatopancreas Health, and Intestinal Microbiome of *Litopenaeus vannamei* in Low-Fishmeal Diet

**DOI:** 10.1155/anu/8883996

**Published:** 2024-12-19

**Authors:** An-Qi Chen, Bao-Yang Chen, Jian Zhong, Zhi-Hong Liao, Xuan-Shu He, Si-Han Lin, Chuan-Ji Fang, Ning Li, Wei Zhao, Jin Niu

**Affiliations:** ^1^State Key Laboratory of Biocontrol, Guangdong Provincial Key Laboratory for Aquatic Economic Animals and Southern Marine Science and Engineering Guangdong Laboratory (Zhuhai), School of Life Sciences, Sun Yat-Sen University, Guangzhou 510275, China; ^2^Zhanjiang Customs, Zhanjiang 534000, China; ^3^Kemin Technology Co. Ltd., Zhuhai 519000, China

**Keywords:** intestinal flora, lipid metabolism, *Litopenaeus vannamei*, low fishmeal diet, lysophospholipid

## Abstract

A 56-day culture experiment was conducted to assess the effects of lysophospholipid added to a low-fishmeal diet on growth performance, hepatopancreas health, and intestinal microbiome of *Litopenaeus vannamei*. Three experimental diets were set up in this study: normal fishmeal positive control diet (20% fishmeal, P), low fishmeal negative control diet (12% fishmeal, N), and low fishmeal + lysophospholipid diet (12% fishmeal with 0.1% lysophospholipid, L). The obtained results proved that *L. vannamei* fed the group N diet could inhibit growth performance (final body weight, weight gain, and specific growth rate), decrease whole-body crude protein, and inhibit hepatosomatic antioxidant capacity and digestive capacity. These adverse effects were significantly alleviated in group L. Compared with group P, the expression of hepatopancreas lipid metabolism genes and the triglyceride content were both increased in group N. The triglyceride level of group L was significantly higher than that of group P but lower than group N. Histological analysis showed that the addition of lysophospholipid could maintain the normal morphology of hepatopancreas and reduce pathological changes such as cell melanosis caused by a low fishmeal diet. In addition, the proportion of dominant colonizers of intestinal flora was unbalanced in group N. In group L, the imbalance was alleviated. In conclusion, the supplementation of lysophospholipid in the low-fishmeal diet of *L. vannamei* improved the weight gain, antioxidant capacity, digestive capacity of hepatopancreas, regulate hepatopancreas lipid metabolism and maintain healthy tissue morphology, and also regulate the intestinal flora structure.

## 1. Introduction

*Litopenaeus vannamei* is an important aquaculture animal with an extensive global market, mainly distributed in tropical waters along the Pacific coast of the United States [1]. It has the advantages of high nutritional value, fast growth rate, and strong environmental adaptability [2].

In the culture of *L. vannamei*, fishmeal is an important feed material and acts as the indispensable high-quality protein source [3]. However, with the expansion of the farming scale, the demand for fishmeal has risen sharply, and there are problems such as high price, uneven quality, and unstable fishmeal supply [4–8]. As a result of the above problems, a major issue in aquatic feed research has always been fishmeal substitution. At present, the common new protein sources in prawn culture are mainly plant protein, single-cell protein, and other animal protein [9]. Soy protein concentrate is a frequently utilized and cost-effective plant protein source; however, it may affect appetite control hormones in animals, resulting in suboptimal feeding outcomes compared to fishmeal [10]. Additionally, plant-based protein sources often exhibit inferior palatability compared to fishmeal, thereby impacting feed efficacy [11]. These problems inhibit the development of plant protein sources in the field of feed.

Current research is concentrated on incorporating functional additives into low fishmeal content feeds to mitigate the negative consequences of fishmeal substitution and enhance feeding efficiency. Emulsifiers, such as phospholipids and bile acids, have been studied as a new type of feed additives in terrestrial biological feeds. It can help emulsify dietary lipids into chylomicrons suspended in the digestive fluid for better absorption [12, 13]. In recent years, the application of emulsifier feed additives has gradually expanded from terrestrial species to aquatic organisms [14]. Studies have found that the use of emulsifiers, such as chenodeoxycholic acid, in low fishmeal diet can promote the growth of *L. vannamei*, regulate lipid metabolism, and maintain the health of hepatopancreas [6].

Lysophospholipid is a kind of emulsifier additive, which is a derivative of lecithin, but its hydrophilic and emulsifying properties are higher than lecithin [15–17]. It can increase the release of monoglycerides and diglycerides by emulsifying lipids [18]. On the one hand, lysophospholipids have an impact on growth performance, feeding efficiency, and antioxidant capacity in terrestrial organisms [19]. On the other hand, it was found that lysophospholipid could improve the utilization rate of fat in feed and regulate lipid metabolism [14]. However, there are few studies on the application of lysophospholipid as feed additives in aquatic invertebrates, and its effect on *L. vannamei* has not been explored before.

In this study, lysophospholipid was evaluated as an additive in low-fishmeal diets to study the effects on growth performance, hepatopancreas health, and intestinal microbiome to provide scientific support for the use of lysophospholipid in *L. vannamei*.

## 2. Materials and Methods

### 2.1. Ethics Statement

All the animal care methods and experimental procedures this study has been approved by the Institutional Animal Care and Use Committee (IACUC), Sun Yat-Sen University. The protocol number is SYSU-LS-IACUC-2023-0020.

### 2.2. Experimental Diets

Experimental diets included three isonitrogenous and isolipidic units, a control group (20% fishmeal, P), a negative control (12% fishmeal, N), and a lysophospholipid group (12% fishmeal with 0.1% lysophospholipid (Kemin Technology Co., Ltd., China), L) (Table 1). Soy protein concentrate was used to maintain the same protein content in experimental diets. Using a sieve with size of 60 mesh, all ingredients were ground and sifted. The ingredients were weighed and blended according to the specified formulations. Afterward, purified water (0.4 L kg^−1^) was incorporated into the blend and mixed thoroughly. The mixtures were transferred to the twin-screw extruder (School of Chemical Engineering, South China University of Technology, Guangdong, China) to extrude the stirred raw materials into strips with a particle size of 1 mm. Then, we transferred the strips to the pelletizer (G-250 shaping pelletizer, Institute of Chemical Engineering, South China University of Technology, Guangdong, China) to make feed strips into experimental feed particles. After granulation, the feeds were transferred to the oven (Guangzhou Weilawei Machinery Equipment Co., Ltd., Guangdong, China), heated at 85°C for 90 min, were taken out and placed in a dry air-conditioned room for natural air drying until the feed moisture drops to about 10%. Feeds were stored at −20°C for later use.

### 2.3. Feeding Experiment

The *L. vannamei* used in the experiment were obtained and cultured in the Sanya Research and Development Center of Tropical Fisheries (Lingshui, China). The farming used seawater extracted from the sea near Lingshui, which was settled and filtered. Before the formal experiment, 360 healthy *L. vannamei* of uniform initial weight (0.93 ± 0.01 g) were selected from the temporary cultured *L. vannamei*. Then, they were allocated randomly to 12 cement tanks (1000 L). Each group was allocated 4 tanks for replication, and 30 shrimps were placed in each tank. Water was changed uniformly and regularly according to the water quality index of regular testing. The tanks were equipped with a gas pipe air stone to maintain uninterrupted aeration for 24 h. The feeding amount was initially set according to 5% of the shrimp body weight, and then adjusted to nearly full feeding by observing the residual bait after feeding. The feeding time was 8:00, 14:00 and 20:00. Dead shrimp, residual bait, feces, and other bottom waste were sucked out by siphon method, and the dead shrimp were weighed and counted in time. During the 8-week culture experiment, the water quality index remained at the following values: water temperature: 29.5–31.5°C (13-WD01-0200, baijun, China); salinity: 35‰ (YHT YHT100B 10060919951479, Yuanhengtong, China); pH: 7.7–8.2; and dissolved oxygen > 6.5 mg/L (YP-SS 100084814627, youyunpu, China). During the feeding trial, a natural day–night cycle was used.

### 2.4. Sample Collection

At the end of the experiment, *L. vannamei* were starved for 24 h. All shrimp in each tank were weighed and counted at the beginning and end of the feeding experiment. Then, the hepatopancreas and intestines of the shrimps were taken for follow-up analysis (4 shrimps/tank), and the whole body of the shrimp was taken for analysis of proximate composition (8 shrimps/tank). At the same time, the posterior intestine samples were obtained under sterile conditions for intestinal microflora analysis (3 mixed samples/group). Samples were frozen in liquid nitrogen, and then stored in a refrigerator at −80°C.

### 2.5. Proximate Composition Analyses

Proximate composition of three diets and whole-body composition of the *L. vannamei* fed with the three diets were determined to follow the standard method described by Association of Official Analytical Chemists (AOAC) [20]. Samples were dried until constant weight was reached to calculate the moisture content. Crude protein was determined on the Dumas nitrogen analyzer (N pro (DT Ar/He Basic), Gerhardt GMBH & CO.KG, Germany). The crude lipid was determined according to the Soxhlet extraction method using an automatic lipid analyzer (Soxtec System HT6, Tecator, Sweden). The ash was detected using a Muffle furnace (M110; In Heraeus, Germany).

### 2.6. Biochemical Analysis

The hepatopancreas were weighed and added ice-cold PBS (1:10 dilution), and then were homogenized until completely broken. The activities of lipase (A054), amylase (C610), chymotrypsin (A080), total superoxide dismutase (T-SOD, S0109), glutathione peroxidase (GPX, A005), aspartate aminotransferase (AST, C010), alanine aminotransferase (ALT, C009), total antioxidant capacity level (T-AOC, A003), malondialdehyde content (MDA, A105), total cholesterol (TC, A111), triglyceride (TG, A110), and low-density lipoprotein cholesterol (LDL-C, A113) in the supernatant of hepatopancreas homogenate and hemolymph were detected by assay kits (Beyotime biotechnology, Shanghai, China; Nanjing Jiancheng Bioengineering Institute, China).

### 2.7. *Quantitative Real-Time Polymerase Chain Reaction* (*qRT-PCR*) *Analysis*

RNAeasy animal RNA isolation kit with spin column (R0026, Beyotime biotechnology, Shanghai, China) was used to extract hepatopancreas total RNA, whose concentration and quality (A260/A280) were determined by Nanodrop 2000 (Thermo Fisher Scientific Inc., USA). Then, we synthesized cDNA by Evo M-MLV reverse transcription kit II (AG11711, Accurate Biology, Hunan). All primer sequences used for qRT-PCR were shown in Table 2. qRT-PCR was completed on a Light Cycler 480II real-time system. The steps were preincubation at 95°C for 10 min, 40 cycles of denaturation for 5 s, annealing for 30 s at 60°C, and then extension for 30 s at 72°C. Expression levels were quantified using the 2^−*ΔΔ*Ct^ method and normalized to *ef-1α*.

### 2.8. Histological Observation

Hepatopancreas were fixed in 4% paraformaldehyde solution, dehydrated in a gradient of ethanol solutions, and then fixed with paraffin embedding. The paraffin embedding sheets were cut into sections with a thickness of about 3.0 μm, and then HE staining was performed with hematoxylin–eosin staining solution. Sections were observed under an optical microscope (Eclipse Ni-E, Japan) and photographed. The images were measured and analyzed using NIS-Elements viewer.

### 2.9. 16S rRNA Gene Sequence Analysis

Genomic DNA was extracted from intestinal contents using a DNA microbiome kit (Qiagen, Germany). Total DNA was evaluated for integrity by 1% AGAR gel electrophoresis, and its concentration and purity were determined using a spectrophotometer (Thermo Fisher Scientific Inc., USA). After purification, the genomic DNA was sent to Majorbio Technology Co., Ltd. (China) for sequencing.

### 2.10. Statistical Analysis Methods

Experimental data were analyzed using SPSS 22.0 (SPSS, Chicago, IL, USA). Results were shown as means ± standard error (SEM). One-way analysis of variance (ANOVA) and Duncan multiple tests were performed for all data among treatment groups, and *p* < 0.05 was considered a significant difference.

## 3. Results

### 3.1. Growth Performance and Feed Utilization

As shown in Table 3, the final body weight (FBW), weight gain rate (WGR), and specific growth rate (SGR) in group N were remarkably lower than those in groups P and L (*p* < 0.05). As compared to groups N and L, group P had a remarkably higher feed conversion rate (FCR) (*p* < 0.05). Survival rate in group P was significantly lower than groups N and L (*p* < 0.05).

### 3.2. Whole Body Composition

According to Table 4, Group N had a significantly lower crude protein concentration than group P. (*p* < 0.05). But neither group L nor group P showed significant differences in crude protein. Meanwhile, moisture, crude ash, and crude lipid contents had no significant differences among groups (*p*  > 0.05).

### 3.3. Hepatopancreas Digestive Enzyme Activities

As shown in Table 5, the activities of lipase, amylase, and chymotrypsin in group N were significantly lower than those in group P (*p* < 0.05), and groups L and P did not differ significantly (*p*  > 0.05).

### 3.4. Hepatopancreas and Hemolymph Biochemical Indexes

Table 6 shows the antioxidant indexes of the hepatopancreas of *L. vannamei*. Compared with group P, T-SOD activity, GPX activity, and T-AOC level of group N were remarkably decreased (*p* < 0.05). MDA content in group L was significantly lower than that in group N (*p* < 0.05). As shown in Table 7, TC content of hemolymph in groups N and L was significantly lower than that in group P (*p* < 0.05). While the hemolymph LDL-C in group P was significantly higher than in both groups, group L was also higher than in group N (*p* < 0.05). P and L groups had significantly lower TG content than N group in hepatopancreas (*p* < 0.05).

### 3.5. Hepatopancreas Gene mRNA Expression

**Figure 1:**
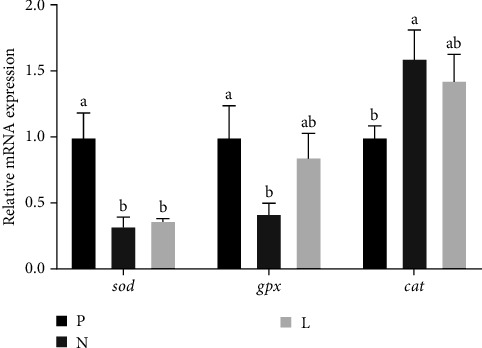
Hepatopancreas mRNA levels of antioxidant enzyme genes of *L. vannamei*. Values are mean ± SEM (*n* = 4). Means with different superscripts are significantly different (*p* < 0.05).

**Figure 2:**
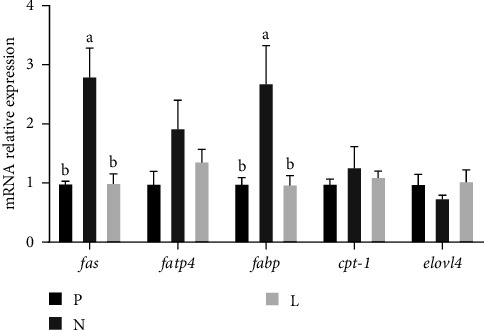
Lipid metabolism mRNA expression in hepatopancreas of *L. vannamei*. Values are mean ± SEM (*n* = 4). Means with different superscripts are significantly different (*p* < 0.05).

The expression level of antioxidant genes is shown in Figure 1. Both groups N and L had significantly lower expression levels of *sod* mRNA than group P (*p* < 0.05). The *gpx* level was not significantly different in groups P and L, but in group N, it was significantly decreased than group P (*p* < 0.05), which was consistent with the results of enzyme activity detection. As compared to group P, group N showed significantly higher expression levels of *cat* (*p* < 0.05).  Figure 2 shows that compared with groups P and L, fatty acid synthase gene (*fas*) and fatty acid-binding protein gene (*fabp*) were significantly increased in group N (*p* < 0.05).

### 3.6. Hepatopancreas Histology

**Figure 3:**
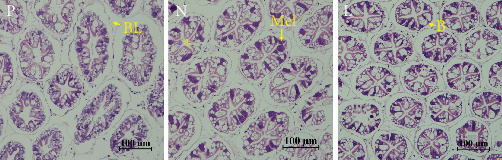
Light microscope section of hepatopancreas morphology of *L. vannamei*. Magnification 200x; B, Blasenzellen cell; BL, basal lamina; Mel, melanization of cells. *⁣*^*∗*^, star shape of the lumen.

As shown in Figure 3, we observed abnormal tissue morphology in the hepatopancreas of group N shrimps. In group N shrimp, the epithelial cells of hepatopancreas showed melanosis, and the B cells became more fragmented.

### 3.7. Intestinal Microbiota Structures

Table 8 shows the diversity index and coverage rate of intestinal flora. Coverage of sample clone libraries of all groups was 0.99. Among all groups, Ace estimator did not show remarkable differences. While group N′s Shannon index was lower than those of groups P and L, group L was noticeably lower than group P (*p* < 0.05).

**Figure 4:**
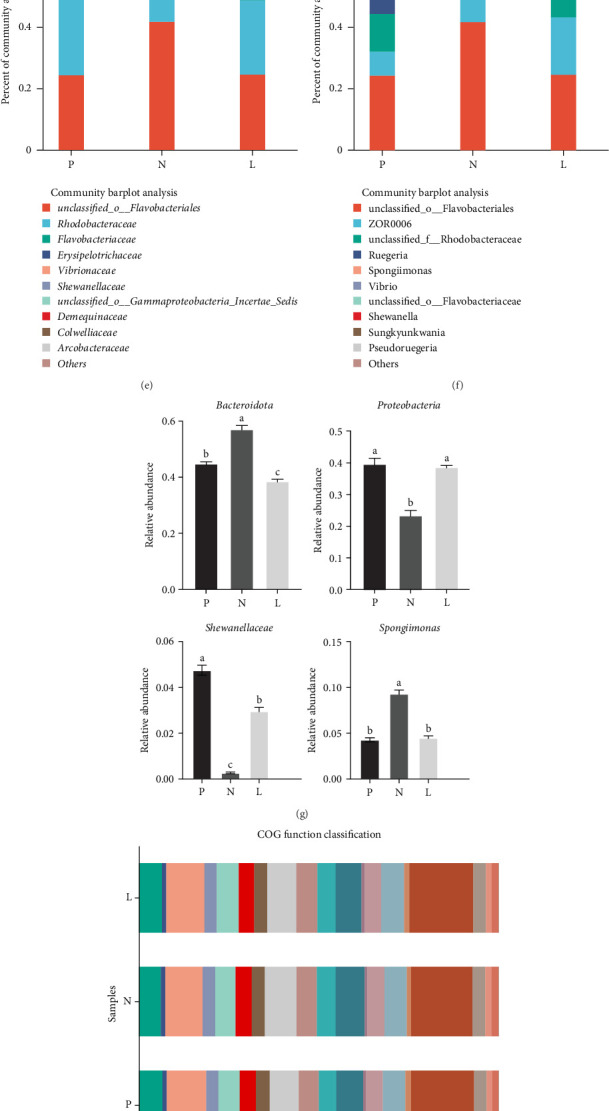
Intestinal microbial community distribution and functional prediction. (A) Venn diagram; (B) percent of community abundance on Phylum level; (C) percent of community abundance on Class level; (D) percent of community abundance on Order level; (E) percent of community abundance on Family level; (F) percent of community abundance on Genus level; (G) Relative abundance with significant differences. Values are mean ± SEM (*n* = 3). Means with different superscripts are significantly different (*p* < 0.05); (H) COG function classification; (I) KEGG function prediction heatmap of pathway.

Figure 4A shows that the number of common species in each group was 176. The number of unique species in group N was 83, which was the largest, followed by 64 species in group L and 54 species in group P. Figure 4B–F shows the microbial community composition of each group at the phylum (B), class (C), order (D), family (E), and genus (F) levels. *Bacteroidota*, *Proteobacteria*, *Firmicutes*, *Actinobacteria*, *Chloroflexi*, etc. were the dominant colonizers at the phylum level. The dominant classes were *Bacteroidia*, *α-Proteobacteria*, *Bacilli*, *γ-Proteobacteria*, etc. At the order level, *Flavobacteriales*, *Rhodobacterales*, *Erysipelothrichales*, *Vibrionales*, and *Alteromonadales* were more abundant. From the family level, the main categories were *Rhodobacteraceae*, *Flavobacteriaceae*, *Erysipelotrichaceae*, and *Vibrionaceae*. Flavobacteriales, Rhodobacteraceae, Ruegeria, and Spongiimonas are the main categories on genus level. Part of the intestinal flora abundance with significant differences (*p* < 0.05) can be seen in Figure 4G. The abundance of *Bacteroidota* and *Spongiimonas* in group N were remarkably higher than those in groups P and L. On the contrary, the group N had significantly lower abundance of *Proteobacteria Shewanellaceae* than the other two groups. The COG functional classification (Figure 4H) has shown that the functions of intestinal microorganisms were mainly energy production and conversion; amino acid transport and metabolism; lipid transport and metabolism, which were closely related to aquatic nutrition. The KEGG function prediction heatmap of the pathway (Figure 4I) shows that the pathways in each group are mainly a variety of metabolic pathways, including carbohydrates, amino acids, energy, vitamins, and cofactors.

## 4. Discussion

Existing studies have shown that lysophospholipid is beneficial to maintaining adequate nutrient supply and activating various functions of the body [19]. The supplementation of lysophospholipid in feed can improve the growth performance in poultry, pigs, and fish [14, 19, 21, 22]. In this study, compared with P group, *L. vannamei* showed lower WG and SGR in group N, and those were improved by the addition of lysophospholipid, which was beneficial to the weight gain of *L. vannamei*. Furthermore, substituting soy protein concentrate for fishmeal or incorporating lysophospholipid into the diet did not result in increased mortality. Additionally, the FCR of groups N and L was lower, suggesting enhanced feed utilization efficiency. This phenomenon may be attributed to the alignment of fishmeal with the natural dietary preferences of *L. vannamei*, leading to higher feed intake in group P. However, the consumed feed may not be fully converted into weight gain. Therefore, it can be inferred that reducing fishmeal supply in feed has the potential to improve production efficiency, which needs functional additives to compensate for the adverse effects of fishmeal deficiency. The body composition data showed that lysophospholipid could promote the deposition of protein in the body. This indicates that lysophospholipid can improve the utilization of dietary nutrients and increase the body weight of *L. vannamei*.

Lysophospholipid can promote the weight gain of *L. vannamei*, which may be due to its effect on the digestive function. As an emulsifier, lysophospholipid contributes to the formation of microemulsion system and increases the contact area between chyme and digestive enzymes [23, 24]. The increase of the contact area between digestive enzymes and substrate can affect their activity, thus promoting the digestion and absorption of feed. Studies have also confirmed that emulsifiers can increase the activities of amylase, protease, and lipase in *Micropterus salmoides* [25], *Cyprinus carpio* [26], and *Salmo trutta caspius* [27]. The experimental results demonstrated a significant reduction in the activities of lipase, amylase, and chymotrypsin in *L. vannamei* following a decrease in fishmeal levels. However, this trend was reversed upon the administration of lysophospholipid, suggesting that lysophospholipid supplementation in a low fishmeal diet aids in maintaining the normal activity of digestive enzymes. These findings are consistent with the previously described growth outcomes.

In addition to producing digestive enzymes, the hepatopancreas of *L. vannamei* are also important antioxidant organs, which can produce a variety of antioxidant enzymes [28]. SOD and GPX are important antioxidant enzymes that can eliminate reactive oxygen species (ROS) in the body [29]. MDA levels can reflect cell damage and assess oxidative stress levels [30, 31]. The results showed that the activities of T-SOD and GPX were significantly reduced after the proportion of fishmeal was reduced, and the antioxidant capacity was weakened. Moreover, MDA levels in group N were significantly increased, indicating *L. vannamei* faced greater oxidative pressure. The addition of lysophospholipid alleviated these adverse effects, and the oxidative pressure of the group L was lower, and the antioxidant enzyme activity was stronger. In the mRNA expression results, the results of *gpx* and enzyme activity were consistent, and the *gpx* mRNA expression in group N was significantly downregulated. CAT is also one of the antioxidant enzymes responsible for clearing ROS and acts as a protective mechanism to avoid tissue damage caused by phagocytosis and ROS [32, 33]. The *cat* in the hepatopancreas was significantly upregulated in group N, perhaps in order to break down the increased hydrogen peroxide in the body to relieve oxidative stress. In summary, lysophospholipid can increase the antioxidant capacity and protect tissue from oxidative stress under the condition of low fishmeal.

As a highly effective emulsifier, lysophospholipid helps dissolve and absorb lipids and reduce the lipid content in the liver to prevent fatty liver [23]. There has been evidence that the total amount of lysophospholipid in aquatic feed can affect their lipid metabolism [19, 34]. TG and TC can reflect the metabolic state of the body. Group N had significantly higher hepatopancreas TG level than group P, which may be the adverse effect of replacing fish meal with soy protein concentrate [35]. However, the group L had remarkably lower TG content rather than group N, which means that lysophospholipid could alleviate the abnormal liver lipid metabolism under the low fishmeal diet.

Hemolymph biochemical parameters serve as indicators of the metabolic and health status of aquatic organisms [36]. Research on *M. salmoides* has demonstrated that the inclusion of lysophospholipid at concentrations exceeding 0.05% in the diet significantly elevates TG levels [37], which corroborated the findings in this study. Furthermore, LDL is a critical lipid droplet responsible for transporting cholesterol and acts as the primary cholesterol carrier within the circulatory system [38, 39]. The role of LDL-C is to transport TC and free fatty acids (FFAs) from peripheral tissues to stem cells for metabolism [25, 40]. The levels of hemolymph LDL-C in groups L and N were significantly lower compared to group P, while LDL-C levels in the hepatopancreas showed a slight increase. This observation suggested that lysophospholipid may facilitate the transport of LDL-C to the hepatopancreas for metabolism. However, the reduction in LDL-C levels could result in impaired energy storage and lipid transport capacity [38]. The notably lower hemolymph LDL-C levels of groups L and N might be negatively influenced by the soy protein concentrate. Nevertheless, compared to group N, group L had a higher level of LDL-C, indicating that lysophospholipid had an alleviating effect on abnormal LDL-C levels in the case of low fish meal.

In addition to the effects on biochemical indicators, lysophospholipid also affects the expression of lipid metabolism genes in the body. In mouse experiments, lysophospholipid can downregulate the expression of *fas* and reduce the accumulation of lipid [41]. FABP is a protein that coordinates lipid transport and signaling in cells, and some subtypes are also closely associated with metabolic and inflammatory pathways [42]. The abnormal expression of *fabp* may be associated with hepatopancreas metabolic diseases. As a rate-limiting enzyme in fatty acid oxidation, CPT-1 also plays an important role in the catabolism of lipids [43]. FATP4 is an acyl-CoA synthetase that is associated with barrier function, and its mutation can lead to lipid homeostasis dysregulation, which leads to fatty liver disease [44, 45]. In this experiment, the mRNA expression levels of *fas* and *fabp* were abnormally upregulated in group N, and the expression levels returned to normal after the addition of lysophospholipid. Low fishmeal diet also affected the m RAN expression levels of *cpt-1* and *fatp4* to a certain extent, and this abnormally high expression could be alleviated by lysophospholipid. These data demonstrated that lysophospholipid has the potential to mitigate the negative impacts of a low fishmeal diet on hepatopancreatic lipid metabolism and sustain lipid homeostasis in *L. vannamei*.

The hepatopancreas morphology of *L. vannamei* was also affected by diet. Hepatopancreas of crustaceans are mainly composed of branched tubules and tubules lined with different types of epithelial cells [33, 46]. It is sensitive to the ingredients in the feed, and its multiple functions, such as absorption, digestion, storage, secretion, and detoxification, may be affected by a poor diet [47]. There are a number of antinutritional factors in soy protein concentrate, such as protease inhibitors, phytic acid, saponins, and allergens [10]. The antinutritional factors impair animals' ability to digest carbohydrates and proteins and cause damage to their hepatopancreas [48]. Consistent with previous results, replacing fishmeal with soy protein concentrate could cause hepatopancreatic tissue lesions in *L. vannamei*. After the addition of lysophospholipid, the hepatopancreas were less damaged, which may be attributed to the improvement of antioxidation capacity and digestive ability.

Intestinal tract is crucial for digestion in *L. vannamei*, and its microbial composition significantly influences nutrient absorption and disease resistance [49]. Most of the gastrointestinal microbiota of *L. vannamei* contain amylase, lipase, and chitinase, which may contribute to the degradation of dietary components [50]. Compared with fishmeal, soy protein concentrate in the diet may also affect intestinal microbes and thus their ability to digest. We conducted a comprehensive analysis of the intestinal flora. The relative abundances of species showed that *Bacteroidetes*, *Proteobacteria*, *Firmicutes*, and *Actinobacteria* acted as the dominant microbial species of *L. vannamei*, which is consistent with the previous studies [51–53]. *Shewanellaceae* has utilizes carbohydrates, and the sharp decrease of this class of flora in group N may affect the carbohydrate metabolism of its prawn intestine [54]. *Spongiimonas* were also frequently reported as the predominant genera in white shrimp gut [55], whose abundance was significantly higher in group N than those in groups P and L. Additionally, the proportion of *Bacteroidota* significantly increased and the proportion of *Proteobacteria* significantly decreased in group N, which reflected the imbalance of intestinal dominant colonizers. From phylum level to genus level, there were significant changes in a certain type of flora in the negative control compared with the positive control, and the relative abundance of some categories in the L group was closer to that in the group P. For example, *Bacteroidota*, *Proteobacteria*, *Shewanellaceae*, *Spongiimonas*, and other bacteria groups in P and L had less differences in relative abundance rather than group L. In comparison to group N, the composition of dominant strains in group L was more similar to that in group P, indicating that lysophospholipid could improve the interference of soy protein concentrate on the predominant colonizers of *L. vannamei* intestinal flora. Furthermore, different diet could lead to different functional predictions of the *L. vannamei* intestinal microbiota. The results showed that intestinal digestion and absorption of fats, amino acids, and other nutrients were affected by intestinal flora, and the addition level of fish meal and lysophospholipid had an impact on it. The KEGG functions prediction of the pathway are mainly metabolic pathways including carbohydrates, amino acids, energy, etc. These results all confirmed the important effects of the intestinal flora on intestinal nutritional function of *L. vannamei*.

## 5. Conclusion

Based on the aforementioned experiments, it was determined that lysophospholipid enhances the weight gain and nutrition value of *L. vannamei* under conditions of a low fishmeal diet. Additionally, lysophospholipid was found to improve digestive enzyme activity and antioxidant capacity in the hepatopancreas, reduce lipid accumulation in the hepatopancreas, and maintain both hepatopancreatic health and the stability of intestinal microbial function. These findings support the potential exploitation and application of lysophospholipid as a functional additive.

## Figures and Tables

**Table 1:** Ingredients of experimental diets (% of dry matter).

Ingredient	P	N	L
Red fishmeal	20	12	12
Dehulled soybean meal	25	25	25
Peanut meal	13	13	13
Chellocken meal	3	3	3
Soy protein concentrate	3	12	12
Wheat meal	24.67	23.07	22.97
Shrimp meal	2.00	2.00	2.00
Beer yeast	2.00	2.00	2.00
Fish oil	0	0.6	0.6
Soybean lecithin	1.5	1.5	1.5
Soybean oil	1.5	1.5	1.5
Vitamin premix^a^	0.5	0.5	0.5
Mineral premix^b^	0.5	0.5	0.5
Choline chloride (50%)	0.2	0.2	0.2
Cholesterol	0.05	0.02	0.02
Ca (H_2_PO_4_)_2_	1.7	1.7	1.7
Lysine (78%)	0.04	0.04	0.04
Ascorbic phosphate ester	0.1	0.1	0.1
Methionine	0.22	0.25	0.25
Threonine	0.02	0.02	0.02
Alginic acid sodium	1	1	1
Lysophospholipid	0	0	0.1
Total	100	100	100
Proximate composition (%)			
Dry matter	89.89	89.68	90.02
Crude protein	39.71	39.95	40.25
Crude lipid	5.20	6.10	5.75
Ash	11.25	11.59	11.09

^a^Composition of multivitamin (kg^−1^ feed): vitamin A, 250,000 IU; Vitamin B2, 750 mg; pyridoxine hydrochloride, 500 mg; vitamin B12, 1 mg; vitamin B1, 500 mg; vitamin K, 250 mg; folic acid, 125 mg; vitamin H, 10 mg; vitamin E, 3750 mg; inositol, 2500 mg; calcium pantothenate, 1250 mg; niacin, 2000 mg; vitamin D3, 45,000 IU; vitamin C, 7000 mg.

^b^Complex mineral composition (kg^−1^ feed): zinc, 4000 mg; potassium, 22,500 mg; iodine, 200 mg; sodium chloride, 2.6 g; copper, 500 mg; cobalt, 50 mg; ferrous sulfate, 200 mg; magnesium, 3000 mg; selenium, 10 mg.

**Table 2:** Primer information.

Gene	Primer sequence (5′-3′)	GenBank no.
*ef-1α*	F: TGGCTGTGAACAAGATGGAC	XM_027373349.1
	R: AGATGGGGATGATTGGGACC	

*gpx*	F: AGAAAGAAGATAAGAGAAGACCCG	XM_027351123.1
	R: TGGTTGGCGGTTGGAATG	

*sod*	F: TGCCACCTCTCAAGTATGATTTC	AB108065.1
	R: TCAACCAACTTCTTCGTAGCG	

*cat*	F: TACTGCAAGTTCCATTACAAGACG R: GTAATTCTTTGGATTGCGGTCA	AY518322.1

*fas*	F: GGATTCTTCCCACCAAGGGG	XM_027367955.1
	R: ACCCAGTTATCACGCCACTG	

*fabp*	F: GCTTTACAGAGGGCGTGAGG	XM_027375657.1
	R: ACTCAATCAACCCTTCCGGC	

*fatp4*	F: CCGACGGGCAAAGCGACTGAACCA	KY271629
	R: TCTATTCCACCAGGTATCTTTATCG	

*cpt-1*	F: CAACTTCTACGGCACTGAT	XM_027361886·1
	R: GTCGGTCCACCAATCTTC	

*elovl4*	F: TTGCAGGGGGCTCTTCTTTC	XM_027382026.1
	R: CACCAGCACTCCCAAACTGA	

**Table 3:** Effects of growth performance and feed utilization of *L. vannamei*.

Items	P	N	L
IBW (g)	0.93 ± 0.01	0.92 ± 0.01	0.93 ± 0.02
FBW (g)	20.92 ± 0.78^a^	18.18 ± 0.18^b^	20.12 ± 0.46^a^
WGR (%)	2140.39 ± 70.44^a^	1869.05 ± 23.16^b^	2062.91 ± 60.86^a^
SGR (% day^−1^)	5.55 ± 0.06^a^	5.32 ± 0.02^b^	5.49 ± 0.05^a^
Survival (%)	90.83 ± 3.70^b^	98.33 ± 1.67^a^	96.67 ± 1.36^a^
FCR	1.45 ± 0.04^a^	1.33 ± 0.03^b^	1.34 ± 0.01^b^

*Note:* FBW (g) = final total wet weight (g)/shrimp number; FCR = total food intake/(FBW − IBW); IBW (g) = initial total wet weight (g)/shrimp number; SGR (% day^−1^) = 100 × [ln (FBW) – ln (IBW)/breeding days; survival rate (%) = 100 × (survival shrimp number)/(initial shrimp number); WGR (%) = 100 × (FBW − IBW)/IBW. Values are mean ± SEM (*n* = 4). Means in the same column with different superscripts are significantly different (*p* < 0.05).

Abbreviations: FBW, final body weight; FCR, feed conversion rate; IBW, initial body weight; SGR, specific growth rate; WGR, weight gain rate.

**Table 4:** Body crude composition of *L. vannamei* (% wet weight).

Groups	Moisture	Crude protein	Crude lipid	Crude ash
P	76.15 ± 0.10	17.41 ± 0.11^a^	1.59 ± 0.05	3.13 ± 0.03
N	76.10 ± 0.07	16.91 ± 0.11^b^	1.45 ± 0.06	3.20 ± 0.04
L	76.41 ± 0.18	17.02 ± 0.15^ab^	1.56 ± 0.05	3.10 ± 0.03

*Note:* Values are mean ± SEM (*n* = 4). Means in the same column with different superscripts are significantly different (*p* < 0.05).

**Table 5:** Digestive enzyme activities of *L. vannamei*.

Groups	Lipase (U/mgprot)	Amylase (U/mgprot)	Chymotrypsin (U/mgprot)
P	15.05 ± 0.79^a^	301.73 ± 22.09^a^	3.02 ± 0.15^a^
N	10.86 ± 1.11^b^	224.20 ± 22.58^b^	2.42 ± 0.10^b^
L	14.62 ± 0.95^a^	281.73 ± 13.18^ab^	2.90 ± 0.14^a^

*Note:* Values are mean ± SEM (*n* = 4). Means in the same column with different superscripts are significantly different (*p* < 0.05).

**Table 6:** Antioxidant capacity of hepatopancreas of *L. vannamei*.

Groups	T-SOD (U/mgprot)	GPX (U/mgprot)	MDA (nmol/mgprot)	T-AOC (U/mgprot)
P	17.16 ± 1.53^a^	201.40 ± 9.86^a^	1.24 ± 0.09^b^	3.05 ± 0.14^a^
N	12.84 ± 0.93^b^	167.71 ± 12.72^b^	1.50 ± 0.08^a^	2.61 ± 0.10^b^
L	16.04 ± 0.80^ab^	198.00 ± 8.02^a^	1.22 ± 0.08^b^	2.96 ± 0.16^ab^

*Note:* Values are mean ± SEM (*n* = 4). Means in the same column with different superscripts are significantly different (*p* < 0.05).

Abbreviations: GPX, glutathione peroxidase; MDA, malondialdehyde content; T-AOC, total antioxidant capacity level; T-SOD, total superoxide dismutase.

**Table 7:** Biochemical indexes of hepatopancreas of *L. vannamei*.

Items	P	N	L
Hemolymph			
AST (U/L)	117.53 ± 3.50	164.9 ± 23.95	155.27 ± 15.97
ALT (U/L)	195.47 ± 17.53	224.62 ± 21.26	222.67 ± 44.38
TG (mmol/L)	0.83 ± 0.18	0.92 ± 0.32	1.09 ± 0.25
TC (mmol/L)	2.87 ± 0.66^a^	1.11 ± 0.08^b^	1.43 ± 0.18^b^
LDL-C (mmol/L)	1.46 ± 0.08^a^	0.33 ± 0.04^c^	0.60 ± 0.02^b^
Hepatopancreas (mmol/gprot)			
TG	0.99 ± 0.26^c^	3.09 ± 0.15^a^	1.97 ± 0.24^b^
TC	0.30 ± 0.02	0.39 ± 0.08	0.39 ± 0.12
LDL-C	5.96 ± 0.72	6.10 ± 0.61	6.43 ± 0.56

*Note:* Values are mean ± SEM (*n* = 4). Means in the same row with different superscripts are significantly different (*p* < 0.05).

Abbreviations: ALT, alanine aminotransferase; AST, aspartate aminotransferase; LDL-C, low-density lipoprotein cholesterol; TC, total cholesterol; TG, triglyceride.

**Table 8:** Index of intestinal microbial diversity.

Groups	Ace	Shannon	Coverage
P	274.07 ± 9.20	3.23 ± 0.07^a^	0.99
N	304.90 ± 1.58	2.44 ± 0.01^c^	0.99
L	298.62 ± 3.25	2.87 ± 0.11^b^	0.99

*Note:* Values are mean ± SEM (*n* = 3). Means in the same column with different superscripts are significantly different (*p* < 0.05).

Abbreviations: Ace, the ace estimator; Coverage, coverage of sample clone libraries; Shannon, Shannon diversity index.

## Data Availability

The datasets will be made available from the corresponding author upon reasonable request.
